# Novel methods for microCT-based analyses of vasculature in the renal cortex reveal a loss of perfusable arterioles and glomeruli in eNOS-/- mice

**DOI:** 10.1186/s12882-016-0235-5

**Published:** 2016-03-02

**Authors:** Daniel S. Perrien, Mohamed A. Saleh, Keiko Takahashi, Meena S. Madhur, David G. Harrison, Raymond C. Harris, Takamune Takahashi

**Affiliations:** Tennessee Valley Healthcare System, Department of Veterans Affairs, Nashville, TN 37212 USA; Department of Orthopaedic Surgery and Rehabilitation, Medical Center East, South Tower, Suite 4200, Nashville, TN 37232 USA; Vanderbilt University Institute of Imaging Sciences, Vanderbilt University Medical Center, Nashville, TN 37232 USA; Department of Pharmacology and Toxicology, Faculty of Pharmacy, Mansoura University, Mansoura, 33516 Egypt; Division of Clinical Pharmacology in the Department of Medicine, Vanderbilt University Medical Center, Nashville, TN 37232 USA; Department of Nephrology, Vanderbilt University Medical Center, Nashville, TN 37232 USA; Vanderbilt O’Brien Mouse Kidney Physiology and Disease Center, Vanderbilt University Medical Center, Nashville, TN 37232 USA

**Keywords:** Micro computed tomography, Glomeruli, Vascular structure, Endothelial nitric oxide synthase

## Abstract

**Background:**

Two-dimensional measures of vascular architecture provide incomplete information about vascular structure. This study applied a novel rigorous method for 3D microCT-based analysis of total and cortical renal vasculature combined with a novel method to isolate and quantify the number of perfused glomeruli to assess vascular changes in eNOS-/- mice.

**Methods:**

Two month old male wildtype and eNOS-/- mice were perfused with heparinized saline followed by radiopaque Microfil. The Microfil-perfused vasculature of excised kidneys was imaged by μCT with an isotropic voxel-size of 5.0 μm. For analysis of renal cortical vasculature, a custom algorithm was created to define the cortical volume of interest (VOI) as the entire volume within 600 μm of the renal surface. Vessel thickness in the whole kidney or renal cortex was analyzed by plotting the distribution of vascular volume at each measured thickness and examining differences between the genotypes at individual thicknesses. A second image processing algorithm was created to isolate, identify, and extract contrast perfused glomeruli from the cortical vessels.

**Results:**

Fractional vascular volume (vascular volume/kidney volume; VV/KV) and Vessel Number/mm (V.N) were significantly lower in eNOS-/- mice vs. WT (*p* < 0.05). eNOS-/- kidneys had significantly fewer perfusable vessels vs. WT in the range of 20–40 μm in thickness. The cortex of eNOS-/- kidneys had significantly lower VV, VV/cortical volume, and V.N, with an increase in the distance between vessels (all *p* < 0.05). The total volume of vessels in the range of 20–30 μm was significantly lower in the cortex of eNOS-/- mice compared to WT (*p* < 0.05). Moreover, the total number of perfused glomeruli was significantly decreased in eNOS-/- mice (*p* < 0.01).

**Conclusions:**

The methods presented here demonstrate a new method to analyze contrast enhanced μCT images for vascular phenotyping of the murine kidney. These data also demonstrate that kidneys in eNOS-/- mice have severe defects in vascular perfusion/structure in the renal cortex.

## Background

Proper renal perfusion is critical for kidney function and many renal diseases result from disruption of blood flow. Alterations in renal blood flow may result from one or more of a number of factors including vascular occlusion, developmental defects in vascular patterning, or loss of vessels secondary to other pathological factors. Hence, proper assessment of renal disease models and treatments often requires measurement of arterial volume and structure in the kidney.

Due to the three-dimensional organization of the vascular tree, two-dimensional assessments of vascular architecture using histology are problematic. Specifically, they are limited to the specific region sampled and the chosen plane of the section. Hence, they cannot accurately determine vessel thickness, volumetric abundance, and connectivity which require three-dimensional, volumetric analytical methods.

Histomorphometric analysis is also destructive and time consuming. While creating 3D reconstructions from micrographs of serial histological sections through the entire kidney is possible, the time and labor required makes such an approach unfeasible in many cases. In contrast, x-ray micro computed tomography (microCT) provides an inherently three-dimensional rendering of microscopic structures based on their attenuation of x-rays. Modern polychromatic microCT systems, available in many laboratories and contract facilities, are capable of generating image volumes the size of whole excised murine kidneys with isotropic voxels as small as 1 μm with 3–5 hour scan acquisition times. However, vasculature and the vascular lumen cannot be delineated from other soft tissues on the basis of native x-ray attenuation, and thus require perfusion with a radiopaque contrast agent for visualization by microCT, as has been described elsewhere [1–7]. Hence, contrast enhanced microCT becomes a powerful tool for visualization of the entire renal vasculature.

paper utilizes eNOS-/- mice to illustrate the utility of novel and comprehensive image analysis methods to detect differences in the contrast-perfusable microvasculature and glomeruli. In the past decades, a body of literature has demonstrated that nitric oxide (NO) produced by endothelial cells through the endothelial nitric oxide synthase (eNOS) plays a major role in regulation of vascular homeostasis including vascular tone, coagulation and platelet aggregation, and inflammation [8] and that eNOS deficiency advances a variety of renal diseases in mice, including diabetic nephropathy and remnant kidney injury [9, 10], indicating a critical role of eNOS in the progression of renal disease. Thus, eNOS -/- mice is widely used to study renal disease. Importantly, recent studies have identified structural defects in the vasculature of eNOS -/- mice, including heart and aorta [11, 12]. Further, two reports have shown that eNOS -/- mice display abnormal renal morphology and progressive renal disease without subjecting to injury [13, 14]. These findings suggest that structural defects may also present in renal vasculature of eNOS -/- mice; however, this issue is poorly addressed.

Previous studies using microCT have described the procedures for renal perfusion with radiopaque contrast and quantification of macro and microvascular volume in the total kidney and the renal cortex. However, the characterizations of renal vasculature in those reports were limited in resolution, volume of interest, the number of morphometric indices examined, vascular structures analyzed and/or by the availability of the microCT technology at that time. Recent advances in widely available polychromatic microCT systems, software, computer power, and analytical algorithms now enable more accurate and rigorous characterization of the renal vasculature and quantification of perfusable glomerular number and density. This paper applies novel approaches to rapidly analyze contrast-perfused vascular structure and glomerular abundance in endothelial nitric oxide synthase (eNOS)-null mice. In doing so, it illustrates a detailed protocol for the analysis of contrast enhanced microCT images of the renal vasculature that, due to its rigorous nature, is capable of detecting a variety of alterations in vascular structure and/or perfusion.

## Methods

### Animal procedures and vascular perfusion

All procedures were approved by the institutional animal care and use committee at Vanderbilt University and were in compliance with AAALAC approved standards. Mice were group housed in standard conditions with 12 h light/dark cycle and free access to food and tap water. Two month old male wildtype C57BL6/J and NOS3-/- mice (B6.129P2-Nos3tm1Unc/J, Stock Number 002684, Jackson Labs, Bar Harbor, Maine) were injected with 10 IU/ml heparin sulfate at least 15 min prior to anesthetization with ketamine and xylazine. Four mice per genotype were analyzed for this study. Under deep anesthesia, the thoracic cavity was opened and a blunt 18-gauge needle was inserted into the left cardiac ventricle. The vena cava was then severed and the mouse vasculature was perfused with approximately 5 ml of 0.9 % saline with 10 IU/ml heparin sulfate. This was followed by perfusion with approximately 8 ml of the radiopaque contrast agent Microfil MV-122 (Flowtech, Inc., Carver, MA). Microfil was prepared immediately prior to starting the infusion procedure at ratio of MV-diluent:MV-compound (5:4) and MV-curing agent (5 % of the total volume). The aorta and vena cava were clamped at the end of perfusion to maintain the perfused volume during curing and the entire carcass was held at 4 °C overnight to promote curing of the Microfil. Both kidneys were excised the following day and placed in 10 % neutral buffered formalin.

### microCT scanning and analysis

The Microfil-perfused vasculature of each kidney was imaged and analyzed by microCT using a μCT50 (Scanco Medical AG, Brüttisellen, Switzerland). Cross-sectional images of the entire kidney were acquired with an isotropic voxel-size of 5.0 μm in a 10.24 mm field-of-view using X-ray source settings of 55 kVp and 200 μA without beam filtering, 1000 projections per rotation, and 700 msec integration per projection. Image acquisition required approximately 2.5 h per kidney.

**Fig. 1:**
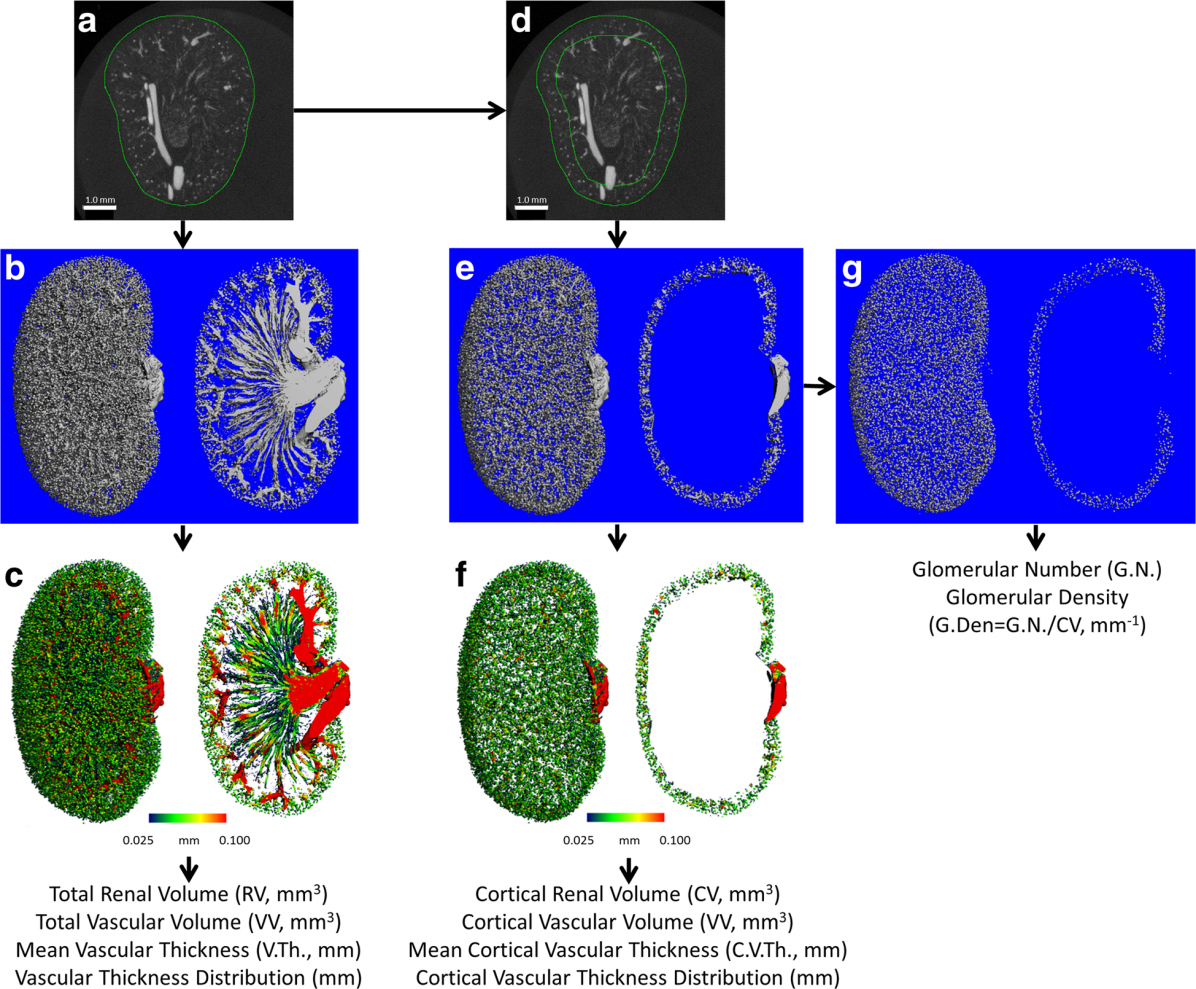
Workflow for defining and analyzing total and cortical renal vasculature and glomeruli by contrast enhanced microCT. **a** The total renal volume was defined using a single contour around outer edge of the kidney. **b** The resulting mask was applied in combination with a grey-scale threshold and three-dimensional Gaussian noise filter to segment perfused vasculature from the total volume and (**c**) the distance transformation method was used to calculate thickness. **d** A mask approximating the renal cortex was created using a computer generated second contour, 600 μm deeper than outer contour (**a**) to exclude the medullary volume from the analysis. **e** and **f** The same threshold, Gaussian noise filter, and distance transformation procedures as in **b** and **c** were then applied. **g** Glomerular structures were then isolated from the three-dimensional segmented cortical vascular image (**e**) using an additional series of transformations to yield an object from which glomerular number and density can be calculated

The entire kidney was selected as the initial volume of interest by hand contouring the outer edge of the kidney in 50–300 slice increments and using an automated ROI interpolation algorithm included in the Scanco Evaluation software to draw contours on the intervening slices. The accuracy of the computer-drawn contours was visually inspected and corrected if necessary. For analysis of renal cortical vasculature, the cortex was defined as the entire volume within 600 μm of the renal capsule (Fig. 1) based on empirical observation of the microCT images. The cortical VOI was created by converting the outer contour to a solid object and performing a three-dimensional peel of 120 voxels (120 voxels x 5 μm/voxel = 600 μm). The “peeled” smaller object (representing the medullary volume) was subtracted from the original object (representing the whole kidney), and the resulting shell (representing the cortical volume), was converted back to a contour file. This “cortical” mask was then used to define the VOI in all further analyses of renal cortical vascular properties.

For both whole and cortical analyses, three dimensional vascular renderings were created by segmenting the soft tissue from contrast perfused vasculature using a grey-scale threshold of 260 and Gaussian noise filter with Sigma of 2.3 and Support of 4. Total volume of the VOI (renal volume (RV) or cortical volume (CV)) and vascular volume (VV) were determined using a direct voxel counting method. Vascular number (V.N), thickness (V.Th), and separation (V.Sp) were determined using the distance transformation method [15]. Since the abundance and total volume of vessels varies according to vessel thickness, thickness data was analyzed by creating histograms representing the vessel volume at each thickness. Mean vascular volume at each vessel thickness was compared across genotypes.

Glomeruli were defined using a combination of erosion and dilation procedures to separate them from afferent and efferent vessels followed by a component labeling algorithm to calculate the volume of each discrete structure and extract only the structures in the range of 45–1000 total voxels (5.625 × 10^−6^ – 1.25 × 10^−4^ mm^3^) (Fig. 1). This range of volumes was chosen based on the glomerular thickness values reported in the literature [1, 16] and empirical observations of the three-dimensional microCT renderings. The use of contrast enhanced microCT presented here is subject to artifacts when determining the true size of objects including the voxel size, noise filter, and x-ray attenuation threshold chosen for segmentation. The settings used here likely resulted in an underestimation of the true glomerular thickness due to volume averaging at glomerular surfaces (accounting for a theoretical reduction of 5 μm in mean thickness), thresholding and noise filtering (possibly reducing the measured thickness by up to 15 μm). The number of remaining discrete objects in the cortical volume of interest was counted as the number of glomeruli (Glom.N).

### Statistical analysis

All data sets were normally distributed and exhibited equal variance. Differences between the two genotypes were analyzed using Student’s t-test and *p* < 0.05 was considered statistically significant. All data are presented as mean +/- standard deviation unless specified otherwise.

## Results

**Fig. 2:**
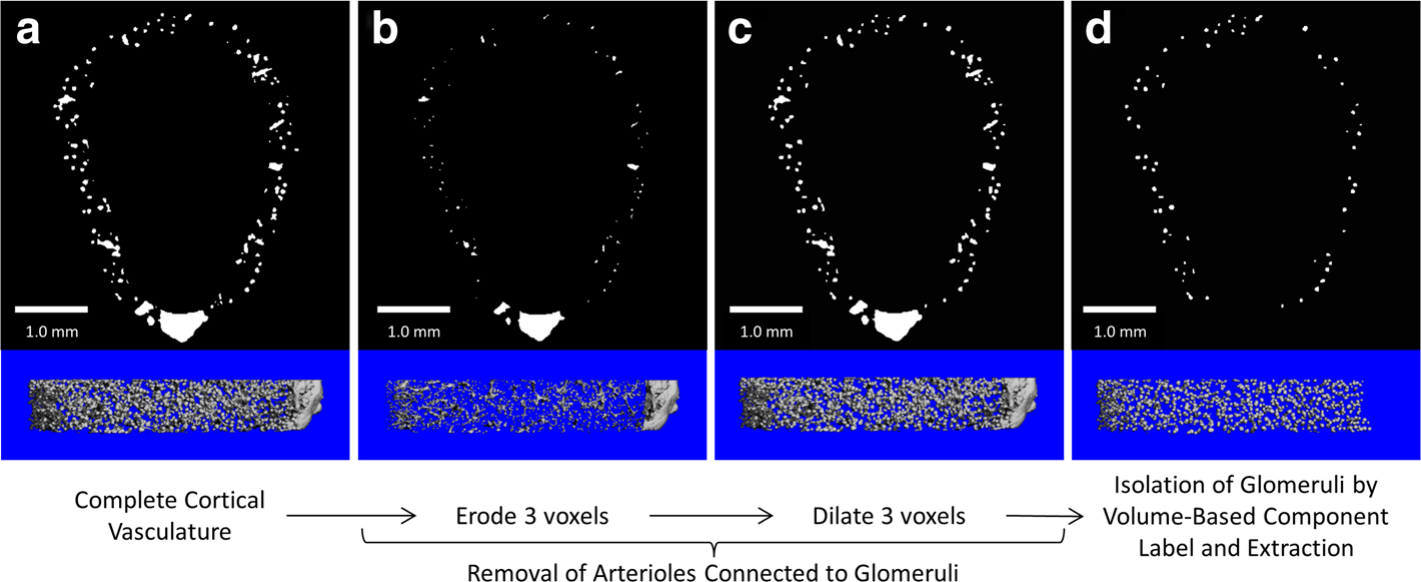
Workflow for isolation of glomeruli from other vascular structures in the renal cortex. A series of erosion, dilation, and component labeling and extraction procedures were used to separate perfused glomeruli from afferent and efferent vasculature. Representative images from each step are shown as both 2-dimensional single slide cross sections (top) and anterior views of a portion of the 3-dimensional objects (bottom). Beginning with the binarized 3-dimensional representation of the cortical vasculature (**a**), three voxels (15 μm) were removed from all surfaces in a 3-dimensional manner using an erosion function resulting in the complete removal of any parts of the object with thickness ≤30 μm, effectively removing any detected capillaries or arterioles connected to the glomeruli (**b**). **c** The original thickness of the now eroded vascular components (**b**) was restored by applying a 3-dimensional dilation of 3 voxels. **d** All individual, disconnected, components of the image in (**c**) were labeled according to the total number of voxels in each object. All objects <45 or >1000 total voxels were removed from the image. The total number of objects remaining, representing the glomeruli, was quantified

Prior to vascular perfusion and sacrifice, the WT and eNOS-/- mice were in generally good health with no obvious problems. Upon visual inspection of the microCT images, it was evident that the selected resolution (a combination of voxel size and signal-to-noise ratio) was not sufficient to accurately segment individual capillaries, but could easily detect glomeruli as their interwoven capillary networks provided greater signal and reduced volume averaging. This result was expected since individual capillaries are only 2–3 times larger than the 5 μm isotropic voxel size used for these studies. Hence, very few voxels encapsulate only capillary volume, while most voxels contain an average of contrast-filled capillary space and non-contrasted tissue around capillaries. While the glomeruli have an apparent thickness that is substantially greater than the afferent and efferent arterioles, their thickness is similar to other, slightly larger vessels in the renal cortex. The overlapping thickness ranges of these structures made it impossible to isolate glomeruli exclusively on the basis of thickness. In contrast, precapillary vessels on the order of >20 μm thick were visible and easily segmented and analyzed on a consistent basis as determined by thickness labeling of the segmented vasculature and the corresponding histograms (Fig. 2).

**Fig. 3:**
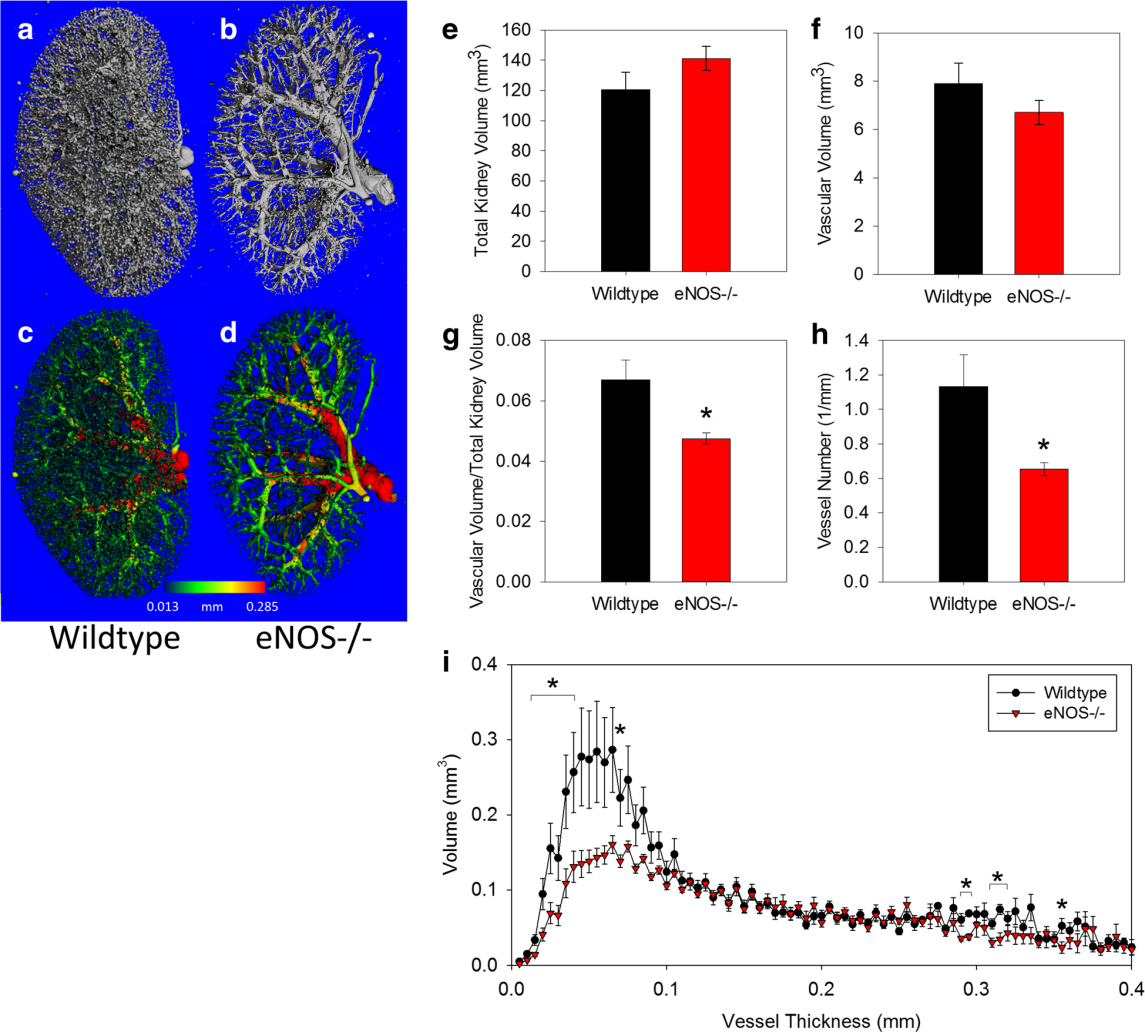
Three-dimensional morphometric analysis of vascular volume and structure in the whole kidney reveals specific deficiencies in the vascular structure of eNOS-/- mice. Representative images of the total perfused renal vasculature illustrate the perfusion deficiency the cortex and smaller vessels in mice eNOS-/- mice (**b** and **d**) compared to WT mice (**a** and **c**). Three-dimensional quantification revealed the deletion of eNOS did not alter (**e**) the total kidney volume or (**f**) the total vascular volume. However, (**g**) the vascular volume/total kidney volume and (**h**) the vascular number in eNOS-/- kidneys was significantly lower than in WT kidneys. **i** Histograms illustrating the total vascular volume at each possible thickness were created and the total volume of perfused vessels at each given thickness was compared between genotypes. This analysis demonstrated a deficit in the number of perfused vessels in the range of 20–80 μm in thickness in the eNOS-/- kidneys compared to WT. All data are mean ± SEM. *p < 0.05 vs. Wildtype by Student’s t-test

Decreased perfusion of contrast into eNOS-/- kidneys compared to those from WT mice was clear upon visual inspection of the 3D reconstructions, which was confirmed by quantification of vascular volume and structure of the whole kidney (Figs. 3a-d). Total kidney volume and total vascular volume were not significantly different between the genotypes (Figs. 3e and f). However, vascular volume calculated as a fraction of the total kidney volume (vascular volume/kidney volume; VV/KV) was significantly lower in eNOS-/- mice compared to WT (Fig. 3g), as was vascular density, represented as the number of vessels/mm (Vessel Number; V.N) (Fig. 3h). To determine which types of vessels were lost and/or obstructed in the eNOS-/- kidneys, vessel thickness in the whole kidney was analyzed by plotting the distribution of vessel abundance at each measured thickness and examining differences between the genotypes at individual thicknesses (Fig. 3i). eNOS-/- kidneys had significantly fewer perfusable vessels that those from WT mice primarily in the range of 20–40 μm in thickness, although significant differences were also found with a few higher thickness measurements (Fig. 3i).

As it relates to vasculature and perfusion, kidney function is primarily determined by factors in the renal cortex where resistance vessels, arterioles, and glomeruli control filtration and reabsorption. Hence, we sought to determine whether the renal vascular phenotype of eNOS-/- mice was more pronounced when the analysis was restricted to the renal cortex. Through visual inspection and measurement of mid-coronal microCT images from multiple kidneys, we determined that the boundary between the renal cortex and medulla was best approximated as 600 μm deep from the kidney capsule and created a volume of interest for each kidney comprising only this volume (Fig. 1).

**Fig. 4:**
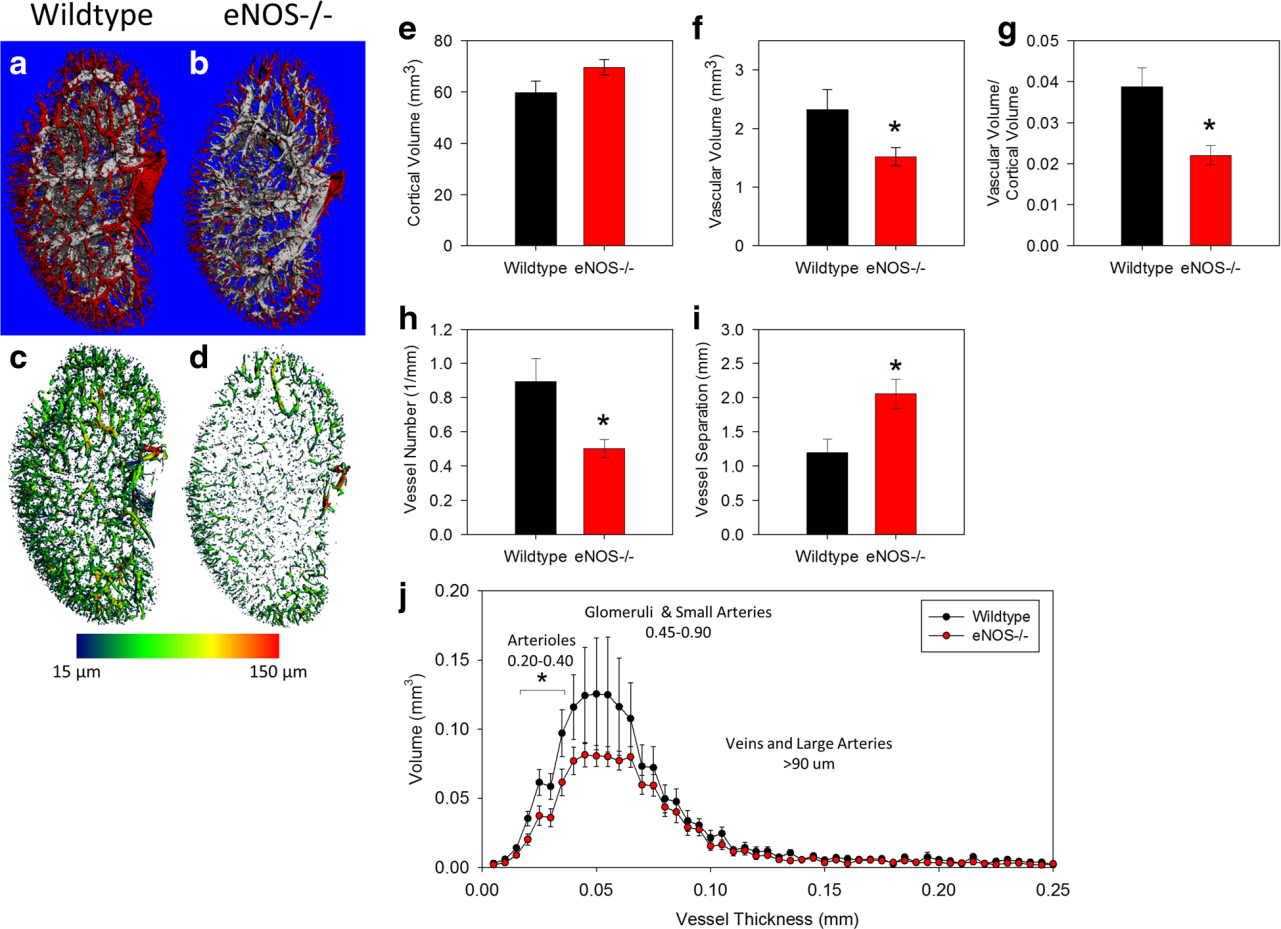
eNOS-/- mice have less perfusable vessels in the renal cortex. Representative images of the renal vasculature in the renal cortex illustrate the perfusion deficiency eNOS-/- mice (**b** and **d**) compared to WT mice (**a** and **c**). Three-dimensional quantification revealed the deletion of eNOS did not alter (**e**) the total cortical volume. However, (**f**) the cortical vascular volume, (**g**) the cortical vascular volume/cortical volume and (**h**) the cortical vascular number in eNOS-/- kidneys was significantly lower than in WT kidneys. **i** Conversely, vessel separation (the mean distance between vessels) was significantly greater in the cortex of eNOS-/- mice. **j** Histograms illustrating the cortical vascular volume at each possible thickness were created and the total volume of perfused vessels at each given thickness was compared between genotypes. This analysis demonstrated a deficit in the number of perfused vessels in the range of 20–40 μm in thickness in the eNOS-/- renal cortex compared to WT. All data are mean ± SEM. *p < 0.05 vs. Wildtype by Student’s t-test

Similar to the whole kidney analysis, three-dimensional renderings of vessels in the renal cortex show a clear decrease in the number and total volume of perfused cortical blood vessels in eNOS-/- kidneys (Fig. 4a-d). Total cortical volume was not significantly different between genotypes (Fig. 4e). However, the cortices of eNOS-/- kidneys had significantly less vascular volume, vascular volume/cortical volume, and vascular number (all *p* < 0.05), with a corresponding increase in the distance between cortical vessels (V.Sp; *p* < 0.05) (Figs. 4f-i). Vessel thickness was again examined as a distribution of the total volume of vessels at each measured thickness. The total volume of vessels in the range of 20–30 μm was significantly lower in the renal cortices of eNOS-/- mice compared to WT (Fig. 4j).

**Fig. 5:**
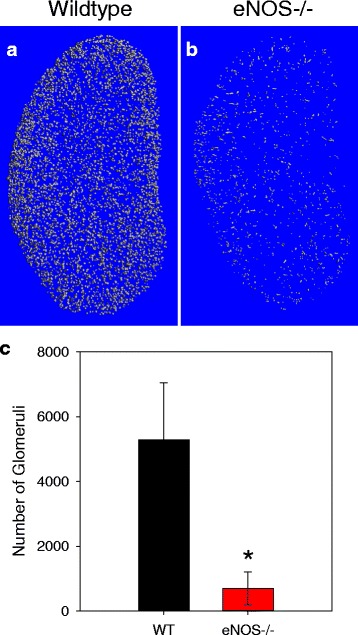
eNOS-/- mice have dramatically fewer perfusable glomeruli. Volumetric quantification of the total number of perfused glomeruli in the renal cortical volumes of interest revealed a dramatic decrease in perfused glomeruli in eNOS-/- mice compare to Wildtype (WT) which could be appreciated both visually (**a** and **b**) and quantitatively (**c**). Data are mean ± SEM. *p < 0.05 vs. Wildtype by Student’s t-test

Isolation of glomeruli from other vascular structures in the cortex produced a number of perfused glomeruli in wild type mice (5290 ± 1758) that was approximately half of the total glomeruli in WT mice obtained using a stereology method (12,283 ± 663) [17]. Comparison between the genotypes revealed a striking difference in the number of perfused glomeruli between WT and eNOS-/- mice (Fig. 5a and b). Quantification confirmed that there were significantly fewer perfused glomeruli in the cortex of the eNOS-/- mice (Fig. 5c).

## Discussion

Precise analysis of renal vascular structure and perfusion is critical to understand the mechanisms of and test potential interventions for numerous renal diseases in animal models. While contrast enhanced microCT imaging and analysis of the renal vasculature has been published previously [1–4], the current data presents an improved analysis capable of detecting changes in arteriole, prearteriole, and glomerular perfusion in the renal cortex. The ability of this approach to focus the analysis on cortical vessels within a specified range(s) of thickness appears to greatly improve the sensitivity of the technique to detect the phenotype of kidneys from eNOS-/- mice. The exclusion of the renal medulla from the volume of interest prevents artifacts created by incomplete filling of the veins which may influence or skew the analysis. Furthermore, this novel method for extracting the perfused glomeruli from the cortical vasculature provides a relatively rapid and cost effective means to detect phenotypic or treatment induced changes in total glomerular number that is not subject to sampling errors in histological assessments.

Applying the vascular thickness distribution to the unique vascular structure of the renal cortex may also provide additional information about the nature of perfusion or structural changes. In the renal cortex, collecting veins are the thickest structures (>100 μm), arterioles are 15–30 μm thick, prearterioles and glomeruli (which appear as solid spheres here) have overlapping thickness in the range of ~30–90 μm, while isolated capillaries and the fine venous structures surrounding collecting ducts are <15 μm. As applied to imaging, the Nyquist theorem states that the smallest structure that can be accurately resolved is in a noise-free image must be at least twice the thickness of the nominal resolution (voxels size). Therefore, at the resolution used here (5 μm voxels; ~12.5 μm resolution) isolated capillaries and fine veins are below the limit of reproducible detection. By excluding structures <15 μm or >100 μm thick, then examining differences in the remaining cortical vascular volume or abundance within a given thickness range, one can surmise whether the observed differences occurred primarily at the arteriolar, prearteriolar, or glomerular levels. Hence, the data presented here demonstrating decreased cortical vascular volume in vessels with thickness 20–40 μm (Fig. 4) suggests a decrease in perfusable renal arterioles in eNOS-/- mice.

This analysis also measured vessel separation in the cortex, a measurement of the mimimum distance between vessels and found a significant increase in the cortex of eNOS-/- mice compared to WT. While this measurement is most commonly applied to the analysis of trabecular bone structure in the literature [18], here it provides insight into the amount of tissue supplied per vessel. As vessel separation increases, the amount of tissue that relies on each vessel increases. Hence, V.Sp may prove useful in detecting areas of potential hypoxia or nutrient depravation in some disease models.

These data clearly demonstrated that microfil-perfusable arterioles and glomeruli are significantly decreased in eNOS -/- mice. Further histological investigation is required to determine if this is due to a reduction in glomerular number or because of vascular restrictions that limit microfil filling of the vessels. While such investigations are beyond the scope of this methodological paper, the fact that eNOS -/- mice at this age show normal serum creatinine levels, kidney weight, and urine albumin excretion [14], suggest that the observed microCT changes may be due to poor perfusion caused by the renal lesions, including focal glomerulosclerosis and renal scaring reported in these mice [13, 14]*.*

Contrast-enhanced microCT analysis of renal vasculature also has a number of limitations which should be carefully considered and controlled, when possible. Since this technique is dependent on reproducible perfusion, extensive practice and confirmation of reproducibility is required before using this to compare phenotypes. Our experience suggests that adequate precision of vascular measurements in wild-type animals can be achieved, although the accuracy of the three-dimensional reconstructions is incomplete, as demonstrated by “breaks” often seen in smaller vessels in the microCT images (data not shown). The acquisition settings and 5 μm voxels used here were sufficient to consistently segment perfused vessels as small as 20 μm in thickness, but the efficiency of detection decreased beginning at about 50 μm thickness (Figs. 3 and 4).

The mean glomerular number in WT mice using the methods presented here are approximately one-half of what is reported with stereological methods. This underdetection is likely due to a combination of incomplete filling and artifacts of the methods used to select the glomeruli including the threshold, noise filter, and volume filtering steps. However, it is important to recognize that these methods are not intended to provide a substitute for stereological quantification of the exact number of glomeruli, but rather a less laborious method to screen for between-group changes in the number of glomeruli that can be perfused with microfil or any other radiopaque contrast agent. Futures studies will benefit from the development and use of contrast agents with lower, more physiological, viscosity.

While new analytical methods are presented here, others have previously employed contrast enhanced microCT and MRI for renal vascular characterization. Beeman and colleagues recently described the use of cationized ferritin as a contrast agent for MRI based quantification of glomerular volume and number in excised human kidneys [19]. This technique was capable of quantifying glomeruli with an approximate 6 % false positive rate. However, the technique required >10 hours of images acquisition time per kidney in a 7 T MRI to achieve the 117 μm isotropic voxel size needed to accurately identify the glomeruli. This technique was previously developed in rats using 9.4 T field and reduced FOV, which still required almost 5 h of acquisition time [20]. Hence, while this approach holds promise for future development, it is currently not feasible for either human or animal studies.

The use of ex vivo contrast enhanced microCT to image renal vasculature has been reported previously [1–3, 5–7, 21]. This technique was first described by Garcia-Sanz in a paper that compared the use of synchrotron and conventional x-ray sources to quantify vascular volume in the renal cortex and regions of the medulla [2]. Their paper reported that while synchrotron-based images were superior in quality microCT images produced from the conventional, polychromatic, x-ray source were of sufficient quality to measure vascular volume in the various anatomic regions and were consistent with histological measurements reported elsewhere [2]. At that time, only the synchrotron microCT was capable of generating images with voxels as small as 6 μm and hence the polychromatic microCT images could not accurately visualize glomeruli. Toyota et al, was the first to report quantification of glomerular volume in 2004. Again, they used synchrotron-based microCT to generate images with 5.6 or 9.6 μm voxels and manually selected 400 glomeruli per kidney to analyze the mean glomerular volume in the 9.6 μm images [1]. While this was a substantial advance, the use of a synchrotron radiation source and time required to manually identify a subset of glomeruli likely prevented additional use of the technique. Another laboratory has reported the use of polychromatic microCT in multiple papers [5–7], however, their analyses were performed in only 12 equidistant two-dimensional slices of the microCT scans rather than in the 3-dimensional renderings. While this was likely due to limited computing power, it nevertheless, reduces the accuracy and precision of the approach. Hence, while laying the groundwork for the current study, previous studies employing contrast enhanced microCT to analyze renal vasculature structures have been limited in scope due to availability of affordable high resolution polychromatic systems, computing power, and analytical software capable of handling these extremely large data sets.

The application of these methods to in vivo images presents an opportunity for future refinement and application that could potentially capture changes in renal blood flow in response to acute stimuli. However, this technique requires the acquisition of images with a nominal isotropic voxel size of approximately 10 μm or less. Given the time, and more importantly, the radiation dose required to achieve this resolution in vivo, repeated in vivo measurements would be challenging with currently available technology. However, since current small animal scanners are marginally capable of performing such scans, this will likely be possible with the next generation of in vivo microCT technology.

## Conclusions

Thanks to advancements in engineering and computer technology, the work presented here represents, to our knowledge, the most rigorous and widely accessible method for complete volumetric analysis of murine renal vasculature published to date. This approach holds the potential to substantially improve renal phenotyping and disease characterization in murine models which may improve understanding of numerous disease models, biological mechanisms, and treatments. The wide availability of affordable polychromatic microCT systems comparable to that used here and powerful parallel processing computer clusters should enable widespread utilization of these techniques.

## Abbreviations

CV: cortical volume
eNOS: endothelial nitric oxide synthase
kV: kilovolts
RV: renal volume
SEM: standard error of the mean
V.N: vessel number
V.Sp: vessel separation
V.Th: vessel thickness
VV: vascular volume
WT: wild type
μA: microamps
μCT: micro computed tomography
